# Sense and sensitivity: can an inaccurate test be better than no test at all?

**DOI:** 10.1136/medethics-2021-107234

**Published:** 2021-04-05

**Authors:** Jonathan Pugh, Dominic Wilkinson, Julian Savulescu

**Affiliations:** 1 Faculty of Philosophy, University of Oxford, Oxford, UK; 2 Oxford Uehiro Centre for Practical Ethics, University of Oxford, Oxford, UK; 3 John Radcliffe Hospital, Oxford, UK3, Oxford, UK; 4 Murdoch Children’s Research Institute, Melbourne, Victoria, Australia

**Keywords:** allocation of healthcare resources, COVID-19, interests of health personnel/institutions, Policy Guidelines/Inst. Review Boards/Review Cttes, public policy

## Abstract

The UK government has put lateral flow antigen tests (LFATs) at the forefront of its strategy to scale-up testing in the coronavirus pandemic. However, evidence from a pilot trial using an LFAT to identify asymptomatic infections in the community suggested that the test missed over half of the positive cases in the tested population. This raises the question of whether it can be ethical to use an inaccurate test to guide public health measures. We begin by explicating different dimensions of test accuracy (sensitivity, specificity and predictive value), and why they matter morally, before highlighting key data from the Liverpool pilot. We argue that the poor sensitivity of the LFAT in this pilot trial suggests that there are important limitations to what we can expect these tests to achieve. A test with low sensitivity will provide false-negative results, and in doing so generate the risk of false assurance and its attendant moral costs. However, we also suggest that the deployment of an insensitive but specific test could identify many asymptomatic carriers of the virus who are currently being missed under existing arrangements. Having outlined ways in which the costs of false reassurance could potentially be mitigated, we conclude that the use of an insensitive LFAT in mass testing may be ethical if (1) it is used predominantly to identify positive cases, (2) it is a cost-effective method of achieving that goal and (3) if other public health tools can effectively prevent widespread false reassurance.

The beginning of widespread vaccination against COVID-19 hopefully heralds the beginning of the end of the pandemic. However, other public health tools such as testing are likely to remain important for some time. A number of different tests can be used to inform public health decision-making; for instance, various kinds of viral detection tests (VDTs) can be used to establish whether an individual is currently infected with the virus, while antibody tests may be used to detect previous infections.^1^

The UK government has recognised the need to increase testing to gain greater control over the virus. The fifth pillar of the UK’s Department of Health and Social Care’s (DHSC) strategy for scaling-up testing highlights the need to build mass-testing capacity and to decentralise testing away from central labs to local hospitals, the workplace and the home.^2^

Moves towards decentralisation raise questions about the standards of accuracy that we require of tests that can be widely deployed. The DHSC has taken a hard line approach in their guidance, explicitly stating that ‘an unreliable test is worse than no test’.^2^ However, recent government policy seems to have ignored or rejected this guidance. Lateral flow antigen tests (LFATs) have already been piloted for mass testing in northern UK cities and for screening health professionals,^3^ and in January 2021, the government announced a national roll-out of community testing using LFATS.^4^ However, preliminary data (published in December 2020) from a pilot of a LFAT in an asymptomatic population suggests that the test missed over half of the positive cases in the tested population.^5^ While commentators have suggested that the data raise the question ‘whether mass screening using a test that performs so poorly is the best use of our limited resources’,^6^ the UK government has nonetheless continued to put LFATs at the forefront of its strategy to scale-up testing, particularly in its approach to reopening schools in March 2021.

Can it be ethical to use an inaccurate test to guide public health measures? We believe that it could be in some circumstances. In order to explain why, it is crucial to first be clear about what LFATs are, and what the pilot data tell us about their accuracy. We shall then explain how the benefits of these tests could be achieved in a way that avoids their most serious moral costs.

## VDTs: LFATs and RT-PCR testing

VDTs are used to identify current infections.^1^ Reverse transcription polymerase chain reaction (RT-PCR) assays have been a central VDT for the UK’s testing strategy.^7^ These assays can be used to identify the presence of SARs-CoV-2 viral RNA through a biochemical process of amplification using enzymes. RT-PCR testing is the standard of care for diagnosing COVID-19 in clinical practice due to its high analytic accuracy.^8^ However, standard RT-PCR tests require processing in centralised laboratories, which is both costly and time-consuming.

There are a range of alternatives that could potentially be used to supplement RT-PCR testing, including whole-genome sequencing, loop-mediated isothermal amplification tests, and LFATs.^9^ LFATs identify SARS-CoV-2 antigens by deploying monoclonal antibodies on a test cartridge that will bind to SARS-CoV-2 antigens in a presented sample. Due to this simpler process, LFATs can be performed at the point-of-care, at a lower cost than RT-PCTs, potentially providing results within 30 mins.

It is important to acknowledge that both RT-PCR testing and LFATs test for infection not infectiousness; they only provide us with information about whether or not a person is infected with the virus, and not how likely they are to transmit it to others.^10^ Although RT-PCR can be used to assess an individual’s viral load, we lack crucial data to establish precisely how viral load relates to infectivity.^11^

The information provided by any VDT is imperfect, since no test is 100% accurate. Accordingly, in order to assess whether LFATs are a suitable supplement to RT-PCR testing, it is important to be clear about different dimensions of test accuracy, and why they matter morally.

## Sensitivity, specificity and predictive value

The first dimension of a VDT’s accuracy relates to how *sensitive* it is to the presence of the virus among infected individuals. A VDT is sensitive when it correctly identifies a high proportion of those who are actually infected with the virus. The more sensitive the test, the less likely it is to incorrectly deliver a false-negative result to an individual who is in fact infected.

False-negative results can have significant moral costs. If the VDT is used to confirm a diagnosis, then a false-negative result may contribute to a missed diagnosis and delay in treatment. Furthermore, individuals who believe that they are not infected (on the basis of a negative result) are less likely to engage in behaviours that will reduce viral transmission. If we assume that at least some individuals with false-negative results will be not only infected but also infectious, then low-sensitivity VDTs will likely increase the risk of viral transmission due to the false reassurance they provide.

A second dimension of a VDT’s accuracy relates to how *specific* it is—this dimension assesses the test’s true negative rate. A VDT is specific when it only identifies a small proportion of people as being infected when in fact, they are not. The more specific a test is, the less likely it is to deliver such false-positive results.

False-positive results also have moral costs.^12^ If the VDT is used to confirm a diagnosis, then a false-positive result may contribute to misdiagnosis. A false-positive result in hospital screening may also lead to the postponement or cancellation of an elective procedure, and at a societal level such results will lead to overestimations of the prevalence of the virus.^12^ It may also result in wasteful further investigation or treatment. Finally, given other public health restrictions, a false-positive test may result in individuals having to self-isolate despite not posing a high risk of transmission. VDTs with low specificity may thus lead to the unnecessary infringement of individual liberties.

However, sensitivity and specificity are not the only dimensions of test accuracy that might interest us. We might also want to know how likely it is that a given result is a true result rather than a false result; that is, we might want to know the test’s *predictive value*.

Crucially, the predictive value of a test is influenced by the prevalence of the virus within the tested population. Suppose you have a test with 99% specificity and 99% sensitivity. This test is highly accurate. However, suppose you use that test in a population of 100 000 people, of which only 1% have the virus. *Ex hypothesi*, in your population, you will have 99 000 uninfected people, and 1000 infected people. If the test is 99% sensitive, you can thus expect it to generate 990 true positives and 10 false negatives in the infected members of the group. With 99% specificity, you can expect the test to identify 98 010 true negatives, and 990 false positives in the uninfected members of the group. So even with a test that is 99% specific and sensitive, there will be as many false-positive results as true positives in this population. So while the negative predictive value of test in this example is 99.99%, its positive predictive value in this population is only 50%, due to the low prevalence of the virus within the population. See table 1 for an illustration of these figures.

**Table 1:** Worked example of a specific and sensitive test with low positive predictive value

*Pop=100 000* *Prevalence=1%* *Sensitivity=99%* *Specificity=99%*	Infected (+)	Not infected (−)	PV
Positive test result (+)	990	990	**Positive PV**=50%
Negative test result (−)	10	98 010	**Negative PV**=99.99%
Total	1000	99 000	

PV, predictive value.

The moral costs of false positive and false negative provide the basis for the DHSC’s claim that ‘an unreliable test is worse than no test’.^2^ Ultimately though, the standards of specificity and sensitivity that we require of VDTs is an ethical judgement. We can only assess this by attending to the moral costs of false-positive and false-negative results, and the benefits of identifying true positive and negative results. For new point-of-care VDTs, the Medicines and Healthcare Regulatory Agency target product profile suggests that the minimum threshold for acceptable test sensitivity is 80% (within 95% CIs of 70%–100%), while the respective threshold for specificity is 95% (within 95% CIs of 90%–100%).^13^ By point of contrast, it has been suggested that RT-PCR assays used in the UK have a sensitivity and specificity of over 95% in laboratory conditions.^12^ (Notably, analytic performance in laboratory settings can differ significantly from real-world operational performance.^12^)

We shall return to the ethical significance of false positive and false negatives below. First though, we shall explain the key data from the Liverpool pilot.

## The Liverpool pilot data

Pilot data from a study of the real-world use of the Innova LFAT for mass testing of asymptomatic participants in Liverpool has suggested that the test had a sensitivity of only 48.89%.^2^ This low sensitivity score led many observers to claim that the Innova LFAT is not fit for the purpose of mass point-of-care testing, due to its high false-negative rate.^6 14^

The Liverpool data include some other interesting nuances. First, despite the low sensitivity score, the test had a negative predictive value of 99.23% in the tested population.^3^ However, the high negative predictive value of the test here is largely due to the fact that there was a low prevalence of the virus in the population—only 45 participants received a positive PCR test result out of the 3026 participants who received a valid result on both a LFAT and PCR test (roughly 1.5%).

The participants in the study also received a PCR test that measured their viral load. Interestingly, the sensitivity of the LFAT test was higher in participants who had a higher viral load—in participants who were found to have the highest viral load following a PCR test, the Innova LFAT’s sensitivity was 85.7%.^4^

Finally, the pilot data suggests that the Innova test had a specificity of 99.93%.^5^ It also had a positive predictive value of 91.67% in this population, despite the relatively low prevalence of the virus.^6^

## Where now for mass testing?

Much of the rhetoric surrounding mass testing has suggested that it can provide reassurance to people that they are not currently infected, and potentially enable access to public spaces.^9^ Indeed, the strategy in the Liverpool study had been to pursue ‘SMART’—Systematic, Meaningful Asymptomatic Repeated Testing. SMART incorporates a three-pronged approach that aims to (1) test to protect (particularly people at highest risk), (2) test to release (eg, people from quarantine earlier) and (3) test to enable (ie, to allow a return to activities).^15^

Further afield, at the time of the initial submission of this paper in January 2021, travellers over the age of 11 have to present evidence of a negative result from an authorised VDT in order to travel from the UK to France. The INNOVA SARS-CoV-2 Antigen Rapid Qualitative Test was one LFAT that was initially authorised for this purpose, but LFAT results are no longer accepted for this purpose.^16^ This is supported by the fact that the Liverpool data regarding the sensitivity of the tests suggest that a negative result can only provide limited assurance that a person is not infected. Indeed, the report of the pilot data states that The Liverpool Health Protection Board decided to pause plans to use the Innova test to enable visitor access to care home settings as a result of the findings.^5^

A test with low sensitivity will provide false-negative results, and in doing so generate the risk of false assurance and its attendant moral costs. The key question then is whether these significant costs could be outweighed by the benefits of using the test. We shall first outline the potential benefits of using LFATs with the degree of accuracy suggested by the Liverpool data, before considering whether it may be possible to diminish the risks and costs of false reassurance that their use might engender.

### The benefits of a specific but insensitive test

RT-PCR is the current gold standard of testing, but such testing in the NHS is largely reserved for symptomatic individuals.^17^ Accordingly, current PCR testing arrangements are poorly suited to identifying asymptomatic carriers. This is a concern, because we know that there are a significant number of asymptomatic carriers of the virus, although estimates vary considerably.^10^ However, we do not have a robust understanding of the contribution of asymptomatic cases to viral transmission^10 18^; indeed, a recent systematic review and meta-analysis found study estimates of the contribution of asymptomatic infection to SARS-CoV-2 transmission ranging from 6% to 69%.^18^

The main tool at our disposal for identifying asymptomatic carriers is tracing the contacts of individuals who have received a positive test result. Notice that even if we assume that the tracing system is functioning effectively, this approach will not be very specific. Suppose X receives a positive PCR test result and has been in contact with Y. There are a number of reasons why X’s contact does not entail that Y will become infected. For instance, X may not have been infectious at the time of contact, or the contact may not have lasted for a sufficient duration to enable infection.

Separately, there have also been well-documented problems with the functioning of the test and trace system in the UK;^19^ an interim report from the National Audit Office highlights the fact that from March to October 2020 the system reached only 66% of the close contacts of index cases. SAGE have advised that an effective test and trace system should reach at least 80% of close contacts.^20 21^ In short, contact tracing to identify asymptomatic carriers may not in practice be effectively identifying a large proportion of asymptomatic carriers, and such an approach will generate a high number of false-positive results.

We are not suggesting that contract tracing should not take place. Rather, we highlight the need to consider alternatives. Obviously, the best alternative would be a test that could be widely deployed, and which is both highly sensitive and specific. However, such an alternative is not currently available. Even though it might be possible to expand PCR testing capacity for the purposes of population screening, such expansion would have considerable financial costs. Moreover, as detailed above, obtaining a PCR test results takes more time than obtaining the result of a LFAT, because of the laboratory processing required by the former kind of testing.

Of course, other things being equal, we should choose to implement a more sensitive test. However, if a cheap, quick and reasonably specific test could be used in a population that would not otherwise have testing, or as a supplement to other forms of positive case identification (like contact tracing), then it will *identify cases that would otherwise have been missed*. That is why a specific but insensitive test can be better than no test. Ensuring that individuals who test positive on an LFAT self-isolate can be justified if the test’s rate of false positives is sufficiently low. For greater certainty, LFATs could be used to triage individuals who are sent for RT-PCR testing.

One important consideration is whether the mass use of LFATs is the best use of resources. To ascertain this, we would need to know how many additional asymptomatic cases mass LFAT testing could be expected to identify, and the cost.^22^ Although LFATs themselves are relatively low cost tests, rolling them out a mass testing programme will require a considerable amount of supplementary resources.

We now turn to consider how the potential costs of an insensitive LFAT test could be mitigated.

### Mitigating false reassurance

The most significant moral cost of false-negative results in mass testing is false reassurance, which may lead individuals to unwittingly spread the virus. The extent to which this moral cost will obtain will depend partly on the context in which the test is deployed, the messaging surrounding the test, and how the results are conveyed. As we now explain, this means that there may be measures that could be employed to mitigate this potential cost of the tests, although they may not succeed in fully addressing this cost.

First, testing strategy could move away from the three-pronged (protect, release, enable) SMART approach. A negative result is more likely to lead a recipient to engage in behaviours that will increase transmission, if the result is used to justify releasing individuals from restrictions affecting those who have not been tested (eg, travelling to France, visiting a care home). Therefore, there is a strong argument against using negative results from an insensitive LFAT to justify exemptions from public health restrictions.

This is one reason why LFATs would not provide a reliable basis for 'immunity passports' of the sort that have been widely discussed as the pandemic has progressed 23 24 Another is that a *true* negative test result on a VDT does not provide any information about the individual’s immunity to future infection; it can only tell us that the individual is not currently infected.

Yet, one could instead use positive results to justify the imposition of further restrictions on individuals (such as self-isolation), without similarly using negative results to justify releasing individuals from other existing restrictions. Indeed, the more stringent the existing restrictions for individuals who have not been tested, the less harm that false reassurance would cause.

Second, such a change in strategy could be accompanied by moving public health messaging away from the three-prongs of SMART, to a two-pronged strategy of protection and case identification. Such testing could be also targeted more specifically at those at greatest risk of exposure; that is, we could justifiably target LFATs predominantly at those who are most likely to have been infected, if the positive results generated by the test are reasonably reliable, and the main purpose of the test on this strategy would be to identify positive cases.

Third, it might also be possible to communicate test results in a manner that clarifies that positive results are robust in a way that negative results are not. This would help to maintain a degree of uncertainty regarding negative results that would serve as a guard against false reassurance. For instance, positive results could be communicated as definite confirmation of infection, while negative results could be framed as inconclusive.

The problem with all of these strategies is that they are difficult public health messages to communicate effectively, and there is a danger that they may reduce willingness to undergo testing. It is thus not clear that they would be sufficient to ensure that the benefits of the test ultimately outweigh the cost of false reassurance.

However, the Liverpool data suggesting that the LFAT used in the study had a higher sensitivity to higher viral load (in conjunction with other LFAT trials suggesting a similar relationship^25^) suggest an alternative way in which further study could help. The moral cost of false reassurance only obtains if we assume that the individuals who receive a false-negative result are also *infectious*; so the crucial question here is what proportion of false-negative results are infectious. We currently lack crucial data to answer this question.^10^ However, if we were to find stronger evidence of the relationship between higher viral loads and increased infectiousness,^7^ this could have two important implications for LFATs that are more sensitive to higher viral loads. First, it would mean that the true positive cases that the tests detect would be in those that are most likely to be infectious. Second, it would mean that many of the the false-negative results would be in individuals who are less infectious. The low sensitivity of a test matters less if false negatives are mostly in individuals who will not go on to transmit the virus.

While we lack crucial data here, this potential feature of LFATs, and the fact that their speed and low cost make regular retesting possible have led some researchers to argue that regulators need to rethink the significance of test sensitivity. Rather than assessing tests on the basis of their sensitivity in one-off uses, Mina *et al* have argued that regulators should assess tests on the basis of their sensitivity when used as part of a regular testing regimen aimed to detect infection in the population.^26^ While there is a need for more data to establish the relationship between viral load and infectiousness, and the sensitivity and cost-effectiveness of non-supervised repeat testing, this highlights one way in which the harms of LFAT testing could be lower if used as part of a targeted testing campaign.

## The normativity of reliability

It is a mistake to think that the reliability of a test is only an objective scientific judgement. Whether a test is reliable enough is determined both by its scientifically determined objective accuracy, but also by whether it is sensitive and specific enough for the desired goals to be achieved. The latter is a value judgement. Whether a test, such as a LFAT, is sensitive enough depends in part on the value we place on liberty and freedom, versus public health. Decisions about where the line is drawn must be informed by judgements about the value of public health as well as the values of liberty, autonomy, justice and non-maleficence.

The value judgments we make in this regard will also be determined by context. For example, if effective treatments are developed, then a false negative is less important. Moreover, as vaccination is rolled-out and we progress towards herd immunity, then a false negative will have a lower chance of transmission. As in all aspects of the pandemic, values and ethics loom large, though they are misrepresented as medical or scientific issues. Issues of test reliability are no different.

The Liverpool pilot data are a significant set-back for what we can expect LFATs to achieve in mass testing. However, it does not necessarily indicate that LFATs should be removed from our public health toolbox—instead, it may change the job that we use them for. It can make sense to use a tool that has a relatively high false-negative rate if it is also highly specific, and if there is a pressing need to identify more infections. It can be ethical to use such a tool when (1) it is used for that prescribed purpose, (2) it is a cost-effective method of achieving that goal and (3) if other public health tools can effectively prevent widespread false reassurance. A test that is accurate in some ways but inaccurate in others can be better than no test at all when it is used wisely.

## Data Availability

(1) The data cited in this study are available in the cited publically available documents.

## Footnotes

Other tests, such as antibody testing can be used to identify previous infections and to provide evidence of potential immunity. For an ethical analysis of such tests.^23^

With 95% CIs of 33.7% to 64.23%. It should be acknowledged that the accuracy performance of the Innova LFAT was measured against RT-PCR– testing as the gold standard.^5^

With 95% CIs of 98.85% to 99.51%.^5^

With 95% CIs of 57.2% to 98.2%.^5^

With 95% CIs of 99.76% to 99.99%.^5^

With 95% CIs of 73% to 98.97%. 5

However, Dinnes *et al* notes some important reasons for doubting the claim that high viral load is correlated with infectivity.^27^
